# Bio efficacy of indigenous biological agents and selected fungicides against branch canker disease of (*Macrophoma theicola*) tea under field level

**DOI:** 10.1186/s12870-018-1445-8

**Published:** 2018-10-10

**Authors:** Mareeswaran Jeyaraman, Premkumar Samuel Asir Robert

**Affiliations:** Plant Pathology Department, UPASI Tea Research Institute, Coimbatore District, Valparai, Tamil Nadu 642127 India

**Keywords:** Biocontrol, Canker size, Chemicals, Field evaluation

## Abstract

**Background:**

Branch canker caused by *Macrophoma theicola* is a major stem disease of tea plants (*Camellia* spp.). In tea plantations, this disease causes crop loss and it is one of the major limiting factor for yield stagnation. In very few instances it causes considerable damage in new clearings (about 3 or 4 years old) and large number of bushes have been killed. As there is no control measures for branch canker disease in south Indian tea plantation, this field study was conducted in naturally infected pruned tea field at UPASI Tea Research Institute (Good Agricultural Practice), Valparai, Tamil Nadu, India.

**Methods:**

The chemical fungicides, biological agents and bio products were evaluated under naturally infected field of seedling plants for two consecutive disease seasons (2014–2015) and there was 11 treatments with three applications. All the treatments were carried out in the time of February–March and October–November (2014–2015). The two set of application was conducted per year. Each set contains eight rounds during the month of February–March as well as October–November (2014–2015). The chemical fungicides, biological agents and commercial bio products were measured as per UPASI- TRF, recommendation viz.*,* COC (50 g/ha and 0.2 g/plot), Companion (20 g/ha and 0.08 g/plot), biological agent of *Bacillus amyloliquefaciens*, *Tichoderma harzianum, Gliocladium virens* and *Beauveria bassiana* (5 kg/ha and 20.8 g/plot) and bio product of Tari (1 L/ha and 4.2 ml/plot) and Tricure (1 L/ha and 4.2 ml/plot).

**Results:**

The present investigation revealed the integrated application of Companion/*Bacillus amyloliquefaciens* showed superior control of branch canker disease followed by the treatment with Companion alone under field condition. Copper oxychloride/*Bacillus amyloliquefaciens* was moderately effective followed by Copper oxychloride. The significantly reduced canker size was recorded with treatment of *Bacillus amyloliquefaciens* followed by commercial organic fungicides of Tari (Organic Tea Special) and Tricure (0.03% Azadirachtin)*.* The least canker size was observed with *Gliocladium virens* followed by *Beauveria bassiana*. Branch canker disease incidence was increased in untreated control plants when compared to treated plants.

**Conclusion:**

Among these 11 treatments, the integrated treatment of companion at rate of 0.08 g and *Bacillus amyloliquefaciens* (20.8 g) showed the most significantly decreased canker size (DPL, 5.76) followed by another treatment with companion (0.08 g) (DPL, 4.11). The moderate reduction of canker size was observed by the treatment with Copper oxychloride (0.2 g)/*Bacillus amyloliquefaciens* (20.8 g) (DPL, 3.05) followed by the treatment of copper oxychloride alone (DPL, 1.74). Therefore, the integrated application of Companion/*Bacillus amyloliquefaciens* proved significantly effective in the management of branch canker disease under the field conditions.

## Background

Tea is the most standard and widely accepted non-alcoholic beverage consumed mainly for its refreshing, mild stimulant effect, medicinal and cosmetic purpose. In commonly cultivated crop shoot contains three leaves and a bud which is used to manufacture different types of tea viz.*,* green tea (non-fermented), black tea (fermented) and oolong tea (semi-fermented) consumed by various group of people according to their tastes. India produces about 1191.1 million kgs of tea from 5, 79,353 ha and in south India around 1, 19,740 ha is grown in Western ghats. In 2015–16 it is estimated that Out of the world’s total production of 5200.3 million kg of tea, 42.9% was produced from China followed by India (22.9%), Kenya (7.7%), Sri Lanka (6.3%), Vietnam (3.2%), Turkey (4.4%), Indonesia (2.5%), Argentina (1.6%), Japan (1.6%), Bangladesh (1.3%), Uganda (1.0%), Malawi (0.7%), Tanzania (0.6%) and Zimbabwe (0.2%). Although tea production in India was more than 1000 million kg, due to high demand of domestic consumption, it is only 13.0% was exported.[1] The first three tea exporting countries are Kenya (25.2%), Sri Lanka (17.2%) and China (18.5%).[1] Crop loss due to canker diseases depends upon severity of pathogen affected infection and the geographical area [2]. Branch canker disease is one of the most serious stem pathogen affected during dry conditions and cause the death of tea bushes more than 40% [3]. All parts of the tea bush viz., foliage, stem and roots are susceptible to diseases and crop loss due to pests and various diseases is around 10–15% [4]. Tea is grown in a wide range of soil types that are acidic in nature and tea soils are latosols in south India [5]. Tea is a monoculture crop and their diseases are classified as leaf, stem and root diseases. The stem diseases are categorized namely collar canker, branch canker, wood rot and thorny stem blight. The branch canker disease is caused by *Macrophoma theicola* which is the major stem disease in south Indian tea plantation. Depends upon the disease severity it causes crop loss and yield stagnations. Previous studies from Taiwan and south-east Asia states that branch canker disease pathogen caused by *Macrophoma theicola* as twig die-back of tea plants and around 40% of tea plants were killed by this disease [4, 6, 7].

The stem diseases such as collar canker (*Phomopsis theae*) and branch canker (*Macrophoma theicola*) creates low yield which are reported from Central Africa [8]. In nursery level conditions, the different tea cultivars are infected by branch canker pathogen *Macrophoma theicola* [9]. Under in vitro conditions, the data available for branch canker disease is very less. Therefore, the initial findings analyzed the branch canker disease characteristic feature, influence of biotic factors viz.*,* pH, temperature and light on pathogen and mycotoxin compounds for disease development in tea plants [10].

The changes in the biochemicals characteristics such as total carbohydrate, reducing sugar and nutrient status of N, P and K were affected by branch canker disease in different tea cultivars under glass house conditions [11]. The entomopathogens *Beauveria bassiana* and *Paecilomyces* sp. used as biocontrol agents as well as biological control of *Bacillus* spp. and *Trichoderma* sp. showed good results against the branch canker disease [12, 13]. The different plant aqueous extracts, chemical fungicide of companion, commercial bio product of Tari and Tricure have been reported as control measures for branch canker pathogen under in vitro conditions [14, 15]. In current situation, there is lack of protection mechanism available for branch canker disease under field level for consecutive disease seasons and it’s very difficult to control this canker during pruning season. The novelty relies in the integrated application of reduced usage of chemical in tea soil as well as by improving the soil health and minimizing the canker incidence level in tea plantation. As far as, in south Indian tea plantation there is no recommendation for branch canker disease. Therefore, the present investigation has been attempted to control branch canker disease with an integrated application of various fungicides and biological control under field level by limiting the usage of chemicals.

## Results

The canker size was reduced significantly after explore of treatments under field condition. Generally, an integrated applications method showed more effectively than straight applications of fungicides. Among the various treatments, the present study revealed that highest reduction of canker size was measured with an integrated treatment of Companion/*B.amyloliquefaciens* followed by straight application of Companion (Table 1). The moderate reduction of canker size was recorded with an integrated spraying of Copper oxychloride/*B.amyloliquefaciens* followed by separate application of Copper oxychloride (Table 1). Furthermore, bacterial strain of *B.amyloliquefaciens* was considerably reduced the canker size followed by the treatment of organic fungicides Tari (Organic Tea Special) and Tricure (0.03% Azadiractin). The least canker size was noted with fungal biocontrol agent of *Gliocladium virens*, *Beauveria bassiana* and *Trichoderma harzianum*. The untreated control plants clearly indicated that canker size was increased than the treated plants (Table 1).

**Table 1:** Evaluation of biological control and fungicides against branch canker disease in tea

S.No	Treatment details	Canker Size (cm)				Disease Protection Level (DPL)
		Pre-Assessment		Post-Assessment		Wound Healing (WH)
		Length in cm (L1)	Width in cm (W1)	Length in cm (L2)	Width in cm (W2)	WH = (L_1 -_ L_2_) + (W_1_- W_2_) in cm
1	Copper oxychloride	7.97	2.53	6.73	2.03	1.74
2	Companion	13.60	4.73	11.19	3.03	4.11
3	*Bacillus amyloliquefaciens*	6.63	3.50	5.56	2.97	1.60
4	Companion/*B.amyloliquefaciens*	14.43	4.10	10.95	1.82	5.76
5	COC/ *B.amyloliquefaciens*	12.97	3.37	10.88	2.41	3.05
6	*Trichoderma harzianum*	13.10	3.00	12.61	2.67	0.82
7	*Gliocladium virens*	9.43	2.70	8.43	2.41	1.29
8	*Beauveria bassiana*	7.50	2.70	6.97	2.29	0.94
9	Tari (Organic Tea Special)	6.23	2.33	5.19	1.96	1.41
10	Tricure (0.03%)	7.87	2.90	7.08	2.33	1.36
11	Control	14.50	3.63	17.49	5.74	−5.10
	CV %	6.32	1.77	6.74	1.76	–
	CD @ 5%	12.31	1.07	11.84	0.89	–

*Mean of three replications

Canker size (cm) was measured before treatment (pre-assessment length and width in cm) and after two years canker size was measured (post-assessment length and width in cm) and their values are given

## Discussion

Generally, there are several integrated disease management reports are available with the combinations of fungicides and biological agents which reduce the disease severity as well as improve the plant health. Earlier findings also reported that combination of bacterial bio agent *Pseudomonas fluorescens* with reduced dose of fungicide to control the *pencillium* rot in pear and combinations of fungicides with yeast have been reported as better integrated application for control the pathogen *Botrytis cinerea* in geranium seedlings [16, 17].

The present study showed that integrated application of Companion/*B.amyloliquefaciens* (DPL, 5.76) was found to provide good results against branch canker disease followed by unaccompanied treatment of Companion (DPL, 4.11) (Table 1). This result was in agreement with the integrated treatment of *Trichoderma harzianum*, *Rhizobium* and carbendazim proved significantly effective in the management of *Sclerotium* root rot of groundnut [18]. Previous report supported that combination fungicide of Companion (Mancozeb 63% + Carbendazim 12%) showed better results against the root rot disease of chilli which is caused by *Sclerotium rolfsii* as well as the superior effect of Companion are proved against botrytis gray mold disease caused by *Botrytis cinerea* in paprika plant [19, 20].

The present result revealed that Copper oxychloride/*B.amyloliquefaciens* (DPL, 3.05) was noticed adequately in reduction of canker size followed by separate treatment of Copper oxychloride (DPL, 1.74) (Table 1). Previous study supported that the moderate reduction of canker size by the combine application of Copper oxychloride + *Bacillus* sp. (Disease protection score, 3.03 cm) [21]. The treatment with contact fungicide Copper oxychloride suggested against the late blight (*Phytophthora infestans*) and early blight diseases (*Alternaria solani*) in potato plant [22].

The present investigation showed that significant reduction of canker size by the treatment with *Bacillus amyloliquefaciens* (DPL, 1.60) followed by the treatment of Tari (Organic Tea Special) and Tricure (0.03% Azadiractin) (Table 1). Our study was in concordance with the previous report that bacterial strain *Bacillus amyloliquefaciens* inhibited the growth and *Sclerotia* production of *Sclerotinia sclerotiorum* at 3rd (4 ml culture filtrate shows 55% inhibition) and 7th (4 ml culture filtrate shows 55% inhibition) days of incubation [23].

The results of bio products obviously indicated that Tari (Organic Tea Special) (DPL, 1.41) and Tricure (0.03% Azadiractin) (DPL, 1.36) reduced the minimal level of canker size (Table 1). The similar observations were noticed with the use of Tari (Organic Tea Special) (69.77 ± 2.83) and Tricure (0.03% Azadiractin) (100.0 ± 0.0) shows higher growth inhibition against branch canker pathogen *Macrophoma theicola* under in vitro conditions at 8% concentration [15].

Our study proved the fungal biocontrol *Gliocladium virens* (DPL, 1.29) showed least level of canker size (Table 1). The result was in negatively correlated with the treatment of *Gliocladium virens* showed better control in reducing collar canker size in tea plants [24]. Whereas, In vitro antagonistic potential of *Trichoderma* sp. and *Gliocladium virens* gave the best control against root pathogens of tea plants [25]. Earlier report supported that integrated disease management to control the basal stem rot of coconut by soil application of *T. viride* [26].

Present study revealed that entomopathogen *Beauveria bassiana* (DPL, 0.94) was recorded least level of canker size followed by *Trichoderma harzianum* (DPL, 0.82) (Table 1). Previous report recommended that *Beauveria bassiana* (64.22) showed higher antagonistic potential against branch canker pathogen *Macrophoma* spp. in tea under in vitro [12]. Most of the reports suggested that entomopathogens of *Beauveria bassiana* and *Lecanicillium* spp. have been identified as biocontrol for the plant disease and soil plant pathogen such as *Rhizoctonia* sp. *Fusarium* sp. and *Pythium* sp. [27–29]. Furthermore, the previous report suggested that *Trichoderma harzianum* and *Trichoderma viride* showed higher inhibitory effect against the pathogen *Poria hypobrunnea* causing stem canker in tea plantation [30]. The observations were made to control thorny stem blight using tea wood pellets colonized by *T. harzianum* and *T. viride* [31]*.* Finally, the whole results are in source with integrated management of branch canker disease in tea and the branch canker disease has been controlled by chemicals, biological control and bio fungicides in tea plantation [32, 33].

## Conclusion

From the above results, it might be concluded that branch canker disease has been successfully recovered from infected field by using integrated application of chemicals and biological control methods. The biological control of the branch canker disease would considerably reduce the fungicide usage in the tea fields and harmful effects of the chemical residues. Our results proved that integrated application of Companion/*Bacillus amyloliquefaciens* controls the branch canker disease well under field conditions. The outcome of this study resulted in evolving an integrated approach to control the branch canker by minimal use of chemical fungicides. As a final point, an integrated application of Companion/*Bacillus amyloliquefaciens* has been identified as superior control of branch canker disease under field condition. In future it could be studied by conducting multi-location field trails.

## Methods

Field experiment of an integrated management of branch canker disease was conducted at UPASI Tea Research Foundation, Tea Research Institute, Valparai located at 10° 23’ North and 77° 0′ East and about 1050 m above MSL. Susceptible of seedling planted in 1961 area at style of planting (1.2 × 1.2 m) and bush population (6800 plants/ha) was conducted. An experiment was carried out in naturally infected pruned tea fields and there was 11 treatments with three replications. Each replication consider as 25 bushes. All the treatments were laid out in randomized block design. The incidence of branch canker disease was assessed at before and after treatment imposed. Treatments and their application details are given in Table 2. The two times of applications per year during February–March and October–November (2014–2015) was studied to the trail plots. The complete applications dosage was measured as per the recommendation schedule of UPASI Tea Research Institute, Valparai, Tamil Nadu, India. The selected fungicides of Copper oxychloride (Fytolan 50% WP) [Bayer Crop Science Ltd.] at 0.2 g and Companion (Carbendazim 12% + Mancozeb 63% WP) [Bayer Crop Science Ltd.] at 0.08 g were explored at 729 ml water volume/25 bush dosage. An indigenous biological control such as *Trichoderma harzianum*, *Gliocladium virens*, *Beauveria bassiana* and *Bacillus amyloliquefaciens* were obtained from repository of Division Plant Pathology, UPASI Tea Research Institute, Valparai, Tamil Nadu, India. Biological control agent was mixed with sterilized talc powder at concentration level of 250 ml culture broth per kg of talc and kept for 2 days of incubation at 25 ± 2 °C. Tricure (0.03% Azadirachtin) at 4.2 ml, Tari (Organic Tea Special) at 4.2 ml and biological agents at 20.8 g were evaluated against the branch canker disease (Table 2). Standard cultural practices spray was done by using a Knapsack low volume sprayer at 5 days interval. The commercial botanical fungicide Tricure (0.03% Azadirachtin) was procured from Margo Biocontrol Pvt., Ltd., Nashik, Maharashtra and Tari (Organic Tea Special) was obtained from TARI BIO TECH, Tanjavur. Branch canker disease protection level was calculated by using following formula. Wound Healing (WH) **=** (L_1_-L_2_) + (W_1_-W_2_). Where **L**_**1**_**- (**Pre-assessment) canker size length in cm, **L**_**2**_**- (**Post-assessment) canker size length in cm, **W**_**1**_- (Pre-assessment) canker size in width (cm), **W**_**2**_- (Post-assessment) canker size in width (cm).

**Table 2:** Treatment details and application dosage of chemicals and biological agents

Treatment details	Dose/ha	Dose/plot	Spraying interval
Copper oxychloride COC (50% WP)	50 g in 10 l of water	0.2 g/729 ml water volume	5 days
Companion (Carbendazim 12% + Mancozeb 63% WP)	20 g/10 L	0.08 g/729 ml water volume	5 days
*Bacillus amyloliquefaciens*	5 kg/ha	20.8 g/729 ml water volume	5 days
Companion / *Bacillus amyloliquefaciens*	20 g/10 L + 5 kg/ha	0.08 g/20.8 g/729 ml water volume	5 days
COC/*Bacillus amyloliquefaciens*	50 g in 10 L of water + 5 kg/ha	0.2 g/20.8 g/729 ml water volume	5 days
*Trichoderma harzianum*	5 kg/ha	20.8 g/729 ml water volume	5 days
*Gliocladium virens*	5 kg/ha	20.8 g/729 ml water volume	5 days
*Beauveria bassiana*	5 kg/ha	20.8 g/729 ml water volume	5 days
TARI (Organic Tea Special)	1000 ml/ha	4.2 ml/729 ml water volume	5 days
Tricure (0.03% Azadirachtin)	1000 ml/ha	4.2 ml/729 ml water volume	5 days
Control	–	Water	5 days

## Abbreviations

COC: Copper oxychloride.
DPL: Disease Protection Level.
GAP: Good Agricultural Practice
L1: Pre-assessment of canker size in length (cm)
L2: Post-assessment of canker size in length (cm).
W1: Pre-assessment of canker size in width (cm)
W2: Post-assessment of canker size in width (cm).
WH: Wound Healing
